# Whole-Genome Sequencing and Mutation Analysis of Two Extensively Drug-Resistant Sputum Isolates of *Mycobacterium tuberculosis* (VRFCWCF XDRTB 232 and VRFCWCF XDRTB 1028) from Chennai, India

**DOI:** 10.1128/genomeA.01173-14

**Published:** 2014-11-13

**Authors:** Lily Therese Kulandai, Dhanurekha Lakshmipathy, Gayathri Ramasubban, Umashankar Vetrivel, Madhavan Hajib Narahari Rao, Sridhar Rathinam, Meenakshi Narasimhan

**Affiliations:** a L&T Microbiology Research Centre, Vision Research Foundation, Chennai, India; b Center for Bioinformatics, Vision Research Foundation, Chennai, India; c Department of Thoracic Medicine, Stanley Medical College, Chennai, India; d Institute of Thoracic Medicine, Chetpet, Chennai, India

## Abstract

We announce the draft genome sequence of two extensively drug-resistant *Mycobacterium tuberculosis* strains, VRFCWCF XDRTB 232 and VRFCWCF XDRTB 1028, isolated from the sputum samples of a patient clinically suspected to have tuberculosis, and we also report novel mutations that confer drug resistance.

## GENOME ANNOUNCEMENT

The global threat of drug-resistant tuberculosis (multidrug-resistant tuberculosis [MDR-TB] and extensively drug-resistant tuberculosis [XDR-TB] has become of great significance in the field of public health. MDR-TB cases threaten the effectiveness of chemotherapy for both the treatment and control of TB and require the use of second-line drugs that are more expensive, toxic, and less effective than first-line anti-TB drugs. Recent reports state that 84 countries have reported at least one XDR-TB case (1). The situation has turned into a pressing demand for rapid genotypic drug susceptibility testing (DST) for the second-line drugs in order to develop efficient regimens for the appropriate treatment of individual cases (2). Therefore, the whole-genome sequencing of XDR-TB strains will aid in a better understanding of the mutation patterns conferring drug resistance, and it provides insights into the exploration of the molecular epidemiology of XDR-TB in a particular geographical location and in the development of newer drug targets to treat tuberculosis.

We announce here the draft genome sequence of two XDR-TB isolates (VRFCWCF XDRTB 232 and VRFCWCF XDRTB 1028) from the sputum samples of two clinically suspected tuberculosis patients. Whole-genome sequencing of two XDR-TB strains was performed using the Ion Torrent PGM platform, similar to that in our previous work (3). The generated sequence reads were adapter trimmed and subjected to reference-based assembly (*Mycobacterium tuberculosis* H37Rv [accession no. NC_000962.3]) using the BWA software 0.6.1-r104. The isolates VRFCWCF XDRTB 232 and VRFCWCF XDRTB 1028 resulted in 156 contigs with a sequence length of 4,369,955 bp and 188 contigs with a sequence length of 4,329,965, respectively, with 63.8× coverage. The assembled sequences were annotated by the NCBI PGAAP (http://www.ncbi.nlm.nih.gov/genomes/static/Pipeline.html). The two strains were further studied for single-nucleotide polymorphism analysis using the SNPsFinder software (http://snpsfinder.lanl.gov/). The single-nucleotide polymorphism (SNP) analysis of VRFCWCF XDRTB 232 revealed novel mutations in the *iniA* (His140Gln) and *rpoB* (Leu1075Pro) genes coding for isoniazid (INH) and rifampin (RIF) drug resistance, respectively. In addition, a novel mutation was observed in the *rpsl* gene (Ala140Val) coding for streptomycin resistance. Regarding second-line antituberculous drugs, novel mutations were observed in the *gyrA* gene (Phe614Ser, Ala668Thr, and Gln384Stop) coding for fluoroquinolone (FQ) resistance, as well as in the *eis* gene (Arg155Stop) coding for aminoglycoside (AMI) resistance. VRFCWCF XDRTB 1028 revealed the presence of novel mutations in the *iniC* (Ala285Val) and *rpoB* (Leu1075Pro) genes coding for INH and RIF drug resistance, respectively. In case of second-line antituberculous drugs, novel mutations were observed in the *gyrA* gene (Phe614Ser, Ala668Thr, and Gln384Stop) coding for FQ resistance, as well as a novel mutation in the *eis* gene (Arg155Stop) coding for AMI resistance. This is the first report of whole-genome sequencing of XDR-TB circulating in the Chennai population from a private research institution in Chennai.

### Nucleotide sequence accession numbers.

The whole-genome shotgun sequences of VRFCWCF XDRTB 232 and VRFCWCF XDRTB 1028 have been deposited at DDBJ/EMBL/GenBank under the accession numbers JNVI00000000 and JQGH00000000, respectively.
